# Editors' Note

**DOI:** 10.5195/ijt.2019.6292

**Published:** 2019-12-12

**Authors:** Ellen R. Cohn, Jana Cason

The *International Journal of Telerehabilitation* (IJT) is a biannual journal dedicated to advancing telerehabilitation by disseminating information about current research and practices. The journal is PubMed indexed, and was recently accepted into Scopus, with indexing to follow.

## ISSUE OVERVIEW

The current issue of the multi-disciplinary *International Journal of Telerehabilitation* (IJT) includes two important clinically based, peer-reviewed articles. Researchers in Singapore describe the use of telerehabilitation to treat frozen shoulder. And, a research team in Australia reports on the delivery of an exercise program designed to improve cardiorespiratory fitness to people dwelling in the community.

This issue also updates readers on the progress made to establish an inter-state licensure compact for the occupational and physical therapy professions. Progress on these efforts can be tracked online for occupational therapy^1^, physical therapy^2^, speech-language pathology^3^, and nursing^4^. It is encouraging that 34 states have enacted NLC legislation and 26 states have enacted the physical therapy compact legislation.

Finally, this issue contains an announcement for a federally supported regional conference –with no limitations on the geographic location of attendees. The MATRC conference program and audience is notably interprofessional.

## CALL FOR SUBMISSIONS

We cordially invite submissions to the Spring 2020 issue by mid- February 2020. IJT accepts original research, case studies, viewpoints, technology reviews, book reviews, and country reports that detail the status of telerehabilitation.

Sincerely,

Ellen R. Cohn, PhD, CCC-SLP, ASHA-F, IJT Editor

Jana Cason, DHSc, OTR/L, FAOTA, Senior Associate Editor

## References

[1] https://www.aota.org/Advocacy-Policy/State-Policy/Licensure/Interstate-Professional-Licensing-Compact.aspx

[2] https://www.fsbpt.org/Free-Resources/Physical-Therapy-Licensure-Compact

[3] https://www.asha.org/advocacy/state/audiology-and-speech-language-pathology-interstate-compact/

[4] https://www.ncsbn.org/nurse-licensure-compact.htm

[5] https://www.library.pitt.edu/e-journals

